# Hypercholesterolemia Causes Circadian Dysfunction: A Potential Risk Factor for Cardiovascular Disease

**DOI:** 10.1016/j.ebiom.2017.04.034

**Published:** 2017-04-27

**Authors:** Makoto Akashi, Ritsuko Matsumura, Takahiro Matsuo, Yuki Kubo, Hiroshi Komoda, Koichi Node

**Affiliations:** a The Research Institute for Time Studies, Yamaguchi University, 1677-1 Yoshida, Yamaguchi 753-8511, Japan; b Department of Cardiovascular Medicine, Saga University, 5-1-1 Nabeshima, Saga 849-8501, Japan

**Keywords:** Hypercholesterolemia, Circadian rhythms, Clock gene, Low density lipoprotein receptor

## Abstract

Hypercholesterolemia is a well-known risk factor for a wide range of diseases in developed countries. Here, we report that mice lacking functional LDLR (low density lipoprotein receptor), an animal model of human familial hypercholesterolemia, show circadian abnormalities. In free running behavioral experiments in constant darkness, these mice showed a prolonged active phase and distinctly bimodal rhythms. Even when the circadian rhythms were entrained by light and dark cycles, these mice showed a significant attenuation of behavioral onset intensity at the start of the dark period. Further, we hypothesized that the combination of hypercholesterolemia and circadian abnormalities may affect cardiovascular disease progression. To examine this possibility, we generated LDLR-deficient mice with impaired circadian rhythms by simultaneously introducing a mutation into *Period2*, a core clock gene, and found that these mice showed a significant enlargement of artery plaque area with an increase in inflammatory cytokine IL-6 levels. These results suggest that circadian dysfunction may be associated with the development or progression of cardiovascular diseases.

## Highlights

• Mice lacking LDLR (low density lipoprotein receptor), a mouse model for hypercholesterolemia, show circadian abnormalities.
• LDLR-deficient mice with a mutation in *Period2*, a core clock gene, show a significant enlargement of artery plaque area.
• *Per2* mutation-induced increase in plasma inflammatory cytokines may cause modification of arteriosclerosis.

Hypercholesterolemia is a common pathology in advanced countries, occurring in 10% to 30% of the population. Here, we report that mice lacking LDLR (low density lipoprotein receptor), an animal model for hypercholesterolemia, experienced chronic circadian abnormalities. Further, we found that LDLR-deficient mice with a mutation in *Period2*, a clock gene, showed a significant enlargement of artery plaque area. Together, our findings indicate that hypercholesterolemia induces circadian abnormalities, leading to a decline in the quality of social life and an increase in the risk of circadian-related diseases, and that circadian dysfunction might affect the development or progression of hypercholesterolemia-related diseases.

## 1 Introduction

Almost all living organisms exhibit circadian rhythms in physiology and behavior, which are driven by the internal circadian clock. The central circadian clockwork consists of a clock gene-driven negative feedback loop of transcription (Partch et al., 2014, Reppert and Weaver, 2001). This cell-autonomous transcriptional feedback loop generates circadian expression of a wide range of numerous genes, which in turn leads to circadian oscillation in diverse physiological processes (Schibler and Sassone-Corsi, 2002). The circadian clock enables maximum expression of genes at appropriate times of the day, allowing organisms to adapt to the earth's rotation. The circadian input for phase adjustment consists of two major pathways. The first is the light input pathway via the hypothalamic suprachiasmatic nuclei (SCN), known as the circadian pacemaker (Welsh et al., 2010). The SCN directly receive photic inputs via the retinohypothalamic tract, and send humoral and neuronal signals to peripheral clocks for systemic adjustment. The second is the feeding input pathway (Bass and Takahashi, 2010, Feng and Lazar, 2012). Temporal feeding restriction changes the circadian phase in peripheral tissues without affecting that in the SCN (Damiola et al., 2000, Stokkan et al., 2001). Thus, the circadian clock is phase-adjusted in response to environmental cues such as light and nutrients in a flexible but phase-dependent manner.

Chronic circadian dysfunction is a significant risk factor for a range of diseases, from sleep disorders to diabetes and cancer (Bass and Takahashi, 2010). A thorough understanding of the mechanisms of circadian dysfunction - a frequent occurrence in the modern lifestyles - is therefore critically important to the prevention of circadian-related diseases. Recently, changes to dietary habits have perpetuated concerns about the adverse effects of chronic metabolic abnormalities on circadian properties. For example, a high-fat diet (HFD) has previously been shown to dampen peripheral clock gene expression rhythms, resulting in a significantly decreased amplitude and a longer free-running period in locomotor activity (Kohsaka et al., 2007). Further studies have revealed that in addition to circadian disruption, an HFD causes large-scale oscillating transcripts de novo, leading to widespread remodeling of biological processes (Eckel-Mahan et al., 2013). Interestingly, time-restricted feeding during an HFD restores oscillations of the circadian clock and their target genes' expression, along with an improvement of metabolic abnormalities including obesity, hyperinsulinemia and hepatic steatosis (Hatori et al., 2012).

Here, we examined the effect of chronic hypercholesterolemia, a common metabolic abnormality in developed countries, on behavioral diurnal rhythms in mice lacking functional LDLR (low density lipoprotein receptor). We further hypothesized that the combination of hypercholesterolemia and circadian abnormalities might contribute to the development and progression of cardiovascular diseases. We examined this by generating *Ldlr* −/− mice with a mutation in *Period2* (*Per2*), a core clock gene.

## 2 Materials & Methods

### 2.1 Animals

*Ldlr* −/− (stock number 002207) and *Per2* m/m (stock number 003819) mice were purchased from Jackson Laboratories (C57BL/6 background), and had been backcrossed with C57BL/6 mice (recipient genome > 99.99% and > 98%, respectively). In all experiments, WT mice were littermates for *Ldlr* −/− mice, but not for *Per2* m/m and *Ldlr* −/− *Per2* m/m mice. *Ldlr* −/−, *Per2* m/m and *Ldlr* −/− *Per2* m/m mice were not littermates. Homozygous *Ldlr* −/− and *Per2* m/m mice were crossed, and the heterozygotes were then crossed again to obtain *Ldlr* −/− *Per2* m/m mice. Genotyping was performed using PCR according to the supplier's instructions. Mice were maintained on a 12:12-h light dark (LD) cycle (light on at 8:00 A.M.), and allowed ad libitum access to food and water. MF (3.6 kcal/g, 61.5% kcal carbohydrate, 25.7% kcal protein and 12.8% kcal fat) was purchased from Oriental Yeast Co. Ltd. (Tokyo, Japan) and used as normal diet (ND), and a high-fat diet (HFD; 3.9 kcal/g, 42.7% kcal carbohydrate, 20.4% kcal protein and 36.9% kcal fat) was purchased from Harlan Laboratories, Indianapolis, IN (TD.94059). Male mice were weaned at 5 weeks of age, and exposed to the ND or HFD at 10–11 weeks of age and maintained with the diet for a period of 20, 24 or 28 weeks. Body weight was measured every 2 weeks. On the night before blood collection, foods were removed from cages. Mouse blood was obtained by cardiac puncture and collected in heparinized tubes. Plasma was collected after centrifugation, and measurements of plasma concentrations of total cholesterol (T-CHO) and triglyceride (TG) were performed in a commercial laboratory (SRL Inc., Tokyo, Japan). Plasma glucose concentrations were measured with a glucometer (Arkray Inc., Japan). Plasma concentrations of IL-6 and insulin were determined using Luminex techniques in accordance with the manufacturer's instructions. All experiments were in accordance with the rules of Yamaguchi University Animal Usage Committee.

### 2.2 Behavioral Analyses

10- to 11-week-old mice were fed with the ND or HFD, and placed in standard mouse cages (W 213 mm, D 324 mm and H 131 mm; CL-0104-2, Japan CLEA Inc., Japan) equipped with infrared sensors to detect locomotor activity. Given the long recording time, we performed experiments under not only single- but also group-housing conditions (three or four male mice together in a single cage) to reduce chronic isolation stress. In the group condition, 25 male mice divided into eight groups were used in each experimental condition. Mice were allowed ad libitum access to food and water, and transferred to clean cages every two months in single-housing conditions or every 2 weeks in group-housing conditions (the actogram is transiently disturbed in the light period). To reduce the frequency of cage cleaning, ALPHA-dri (Shepherd Specialty Papers, Inc., U.S.A.), made from alpha cellulose and having high absorbency, was used as animal bedding. For the first several days of activity recording, mice were maintained on a 12:12 LD cycle. After that, animals were exposed to light and dark (LD) or constant darkness (DD). Locomotor activity was measured using infrared thermal sensors, and the spontaneous locomotor activity data obtained were analyzed using Clock Lab software (Actimetrics Inc.). The free-running period and amplitude (Qp value) of circadian behavioral rhythms was calculated with a chi-square periodogram (Hirano et al., 2013, Sokolove and Bushell, 1978). To facilitate the comparison of activity levels over the measurement period, average 6-min bin activity of several-day periods was calculated, and shown as a line graph. Onset intensity was calculated as the peak level of behavioral activity just after the start of dark phase. More specifically, average 6-min bin activity was calculated for the indicated periods, and the maximum 6-min bin value within 2 h after light-off was defined as onset intensity. Data are shown as the average onset intensity with SE (*n* = 8 mice). No mice showed the maximum 6-min bin activity before light-off.

### 2.3 Aortic Analysis

Mice were euthanized 20, 24 and 28 weeks after exposure to the HFD, and the atherosclerotic lesions were analyzed as follows. After mice were anesthetized with pentobarbital sodium (80 mg/kg ip; Abbot Laboratories, Abbott Park, IL), the aorta was perfused with normal saline containing heparin (10 U/ml). Aortic samples excised from the aortic sinus were then dissected free from surrounding tissues, opened longitudinally, and pinned onto a silicon-coated dish. A 0.5% Oil Red O stock solution was prepared in 100% isopropanol and filtered through a 0.2-μm filter. A working solution was prepared by mixing three volumes of stock solution and two volumes of water, which was allowed to stand for 10 min and then filtered through a 0.2-μm filter. Aortic samples were fixed in 10% neutral-buffered formalin for 15 min at room temperature, and then washed with 50% isopropanol three times. Samples were stained in the working solution at room temperature for 15 min, then washed with 50% isopropanol for 5 min. Aortic samples were kept in 10% neutral-buffered formalin for imaging. Image analysis was performed using the ImageJ Software (NIH, Bethesda, MD). The amount of aortic lesion formation in each animal was measured as the percentage of lesion area per total area of the aorta.

## 3 Results

### 3.1 Hypercholesterolemia Attenuates Behavioral Onset Intensity in Light-Dark Cycles

**Fig. 1:**
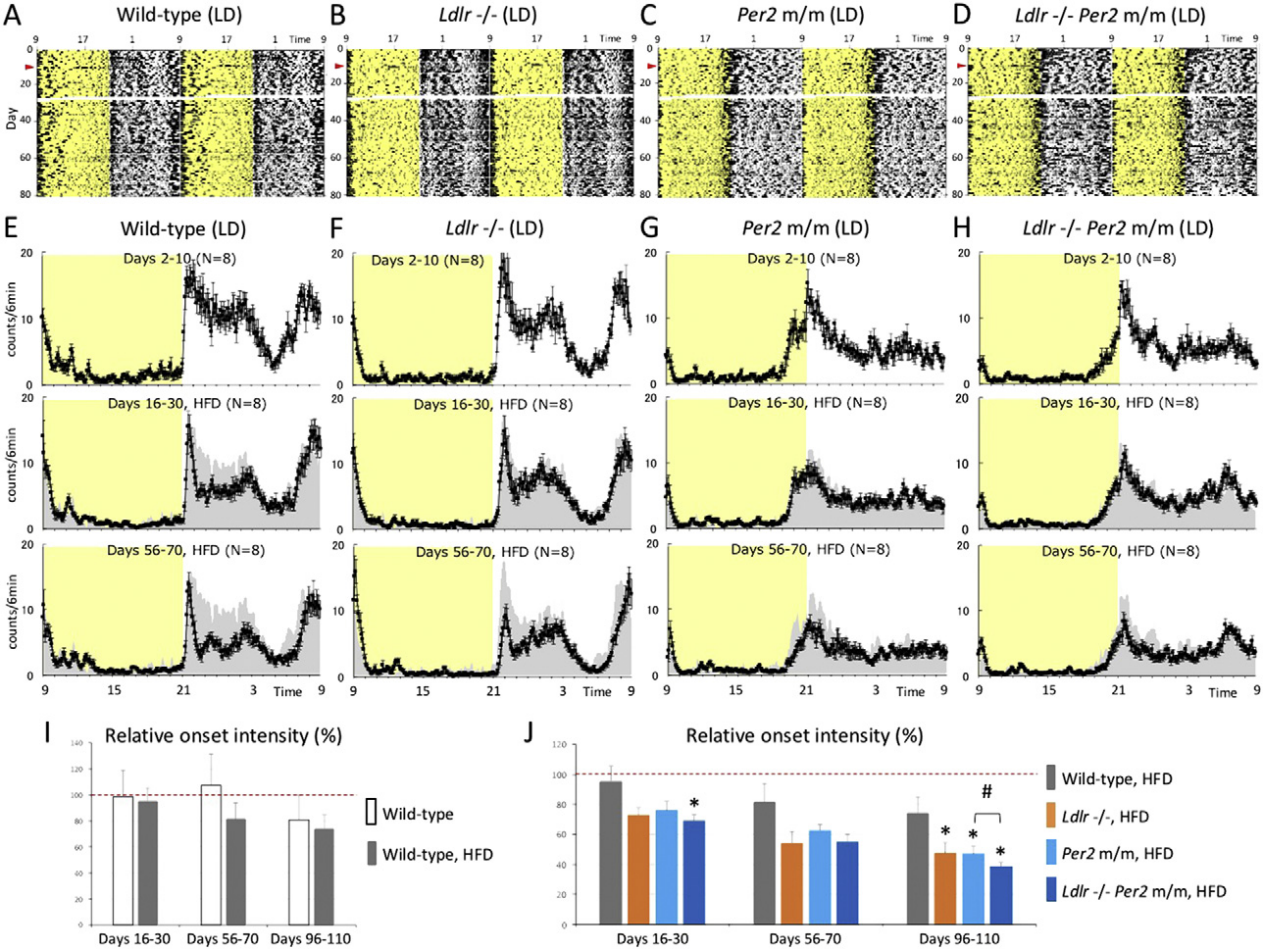
Effect of hypercholesterolemia on behavioral rhythms in light and dark. (A, B, C and D) Continuous monitoring of the locomotor activity of singly-isolated mice was performed using an infrared sensor in light and dark. Representative raw activity data of wild-type, *Ldlr* −/−, *Per2* m/m and *Ldlr* −/− *Per2* m/m mice are shown as a double plot. Exposure to an HFD was started on day 11 (red arrowhead). Data at days 27 and 28 were not recorded due to an instrumental communication error. Yellow shadows indicate light periods. (E, F, G and H) Average 6-min bin activity was calculated for several-day periods on days 2–10 (before exposure to the HFD), 16–30 (shortly after exposure to the HFD), and 56–70, and shown as a line graph. Data are the average ± SE of eight mice. Yellow shadows indicate light periods. To facilitate visual comparison before and after HFD treatment, gray silhouettes represent data at days 2–10 (normal chow diet). (I and J) Onset intensity was calculated as the peak level of behavioral activity just after the start of dark phase. The onset intensity of all mice on days 2–10 was set to 100% (red dashed line), and the graph therefore represents an attenuation rate of the onset intensity with regard to temporal change after exposure to the HFD. The data indicate the average ± SE (*n* = 8). A *t*-test (vs. control) was performed, and statistical significance was defined as *P* < 0.05. Asterisks indicate a significant difference from wild-type (+ HFD) mice, and pound signs indicate a statistically significant difference between *Per2* −/− and *Ldlr* −/− *Per2* m/m mice.

**Fig. 2:**
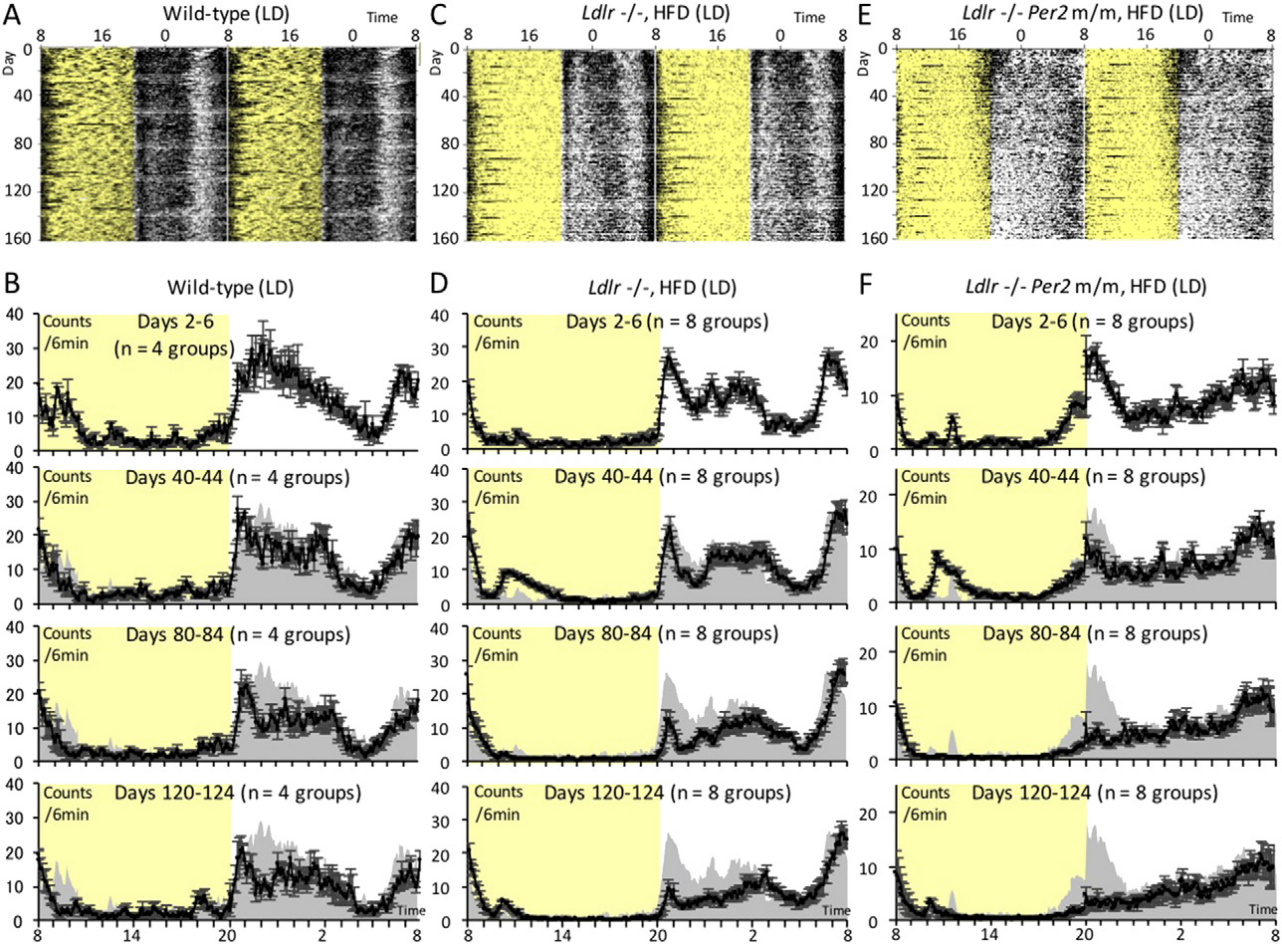
Effect of long-term hypercholesterolemia on behavioral rhythms under light-dark and group-housing conditions. (A to F) Long-term monitoring of locomotor activity was performed over a period of up to about 160 days in light and dark. Representative raw activity data of wild-type mice, *Ldlr* −/− mice (+ HFD) and *Ldlr* −/− *Per2* m/m mice (+ HFD) are shown in (A), (C) and (E), respectively. Average 6-min bin activity was calculated for five-day periods at intervals of about 40 days (days 2–6, 40–44, 80–84 and 120–124), and shown as a line graph. The average ± SE (*n* = 4 or 8 groups) are shown in (B), (D) and (F), respectively. Yellow shadows indicate light periods. To facilitate visual comparison before and after HFD treatment, gray silhouettes represent data at days 2–6 (normal chow diet).

To examine the effect of hypercholesterolemia on circadian rhythms, we performed behavioral experiments on mice lacking functional LDLR, an animal model for human familial hypercholesterolemia. These mice showed chronic high blood cholesterol, a leading cause of arteriosclerosis-diseases (Ishibashi et al., 1993). We used an infrared sensor to record the locomotor activity of *Ldlr* −/− mice fed an HFD in single-housing and light-dark conditions (Fig. 1). Wild-type mice fed a normal diet or HFD were used as controls. To investigate whether hypercholesterolemia affects locomotor activity through the core clock machinery, we generated double *Ldlr* −/− *Per2* m/m mice; the *Per2* mutation leads to a deletion in an 87 amino acid sequence in the PER2 protein (Zheng et al., 1999). *Per2* m/m and *Ldlr* −/− *Per2* m/m mice were also fed the same HFD in single-housing and light-dark conditions. Since long-term isolated animals under single-housing conditions have been shown to exhibit aggressive, anxiety-like behaviors, and abnormal locomotor activity (Blanchard et al., 2001, Rodgers and Cole, 1993), in addition to single-housing conditions, we conducted locomotor experiments under group-housing conditions to reduce chronic isolation stress and to confirm the reproducibility of the results obtained under single-housing conditions (Fig. 2).

Under single-housing conditions, mice were exposed to the HFD on day 11 (Fig. 1A–D). The average activity level was calculated for several-day periods at three time-points: days 2–10 (before exposure to HFD), days 16–30 (shortly after exposure to HFD), and days 56–70 (long-term exposure to HFD) (Fig. 1E–H). During days 16–30, mice under all experimental conditions showed similar circadian behavior to that at days 2–10 (normal chow feeding). Consistent with a previous report (Kohsaka et al., 2007), wild-type (+ HFD) mice showed reduced behavioral activity during the mid-dark phase, while activity in other phases was not affected. The circadian pattern of wild-type (+ HFD) mice during days 16–30 was similar to that of *Ldlr* −/− mice during days 2–10 (normal chow feeding), which showed a higher total blood cholesterol concentration compared to wild-type (− HFD) mice. As reported in the original study on targeting disruption of the *Per2* gene, *Per2* m/m and *Ldlr* −/− *Per2* m/m mice showed an earlier shift in onset of activity (Zheng et al., 1999). Prolonged monitoring showed that behavioral onset intensity at the start of the dark period was significantly suppressed with time in *Ldlr* −/− (+ HFD) mice. This was not observed in wild-type (+ HFD) mice, whose activity onset intensity was similar to that of wild-type (− HFD) mice (Fig. 1I and J). The suppression in onset intensity in *Ldlr* −/− (+ HFD) mice was more drastic in mice under group-housing conditions (Fig. 2). These results indicate that the suppression in onset intensity may be attributed to more severe disruption of the circadian system by hypercholesterolemia. Consistent with this hypothesis, *Per2* m/m mice with the wild-type *Ldlr* allele (+ HFD) showed a similar attenuation of onset intensities to that of *Ldlr* −/− (+ HFD) mice, suggesting that the suppression of behavioral onset in *Ldlr* −/− (+ HFD) mice might occur through dysfunction of the circadian machinery. We confirmed similar onset intensity attenuation and a statistically significant but small synergistic effect in *Ldlr* −/− *Per2* m/m (+ HFD) mice under both single and group-housing conditions, indicating that *Ldlr* knockout and *Per2* mutation caused onset intensity attenuation through dysfunction of the same pathway, namely the circadian pathway (Fig. 1I and J).

### 3.2 Hypercholesterolemia Significantly Affects Behavioral Rhythms in Constant Darkness

**Fig. 3:**
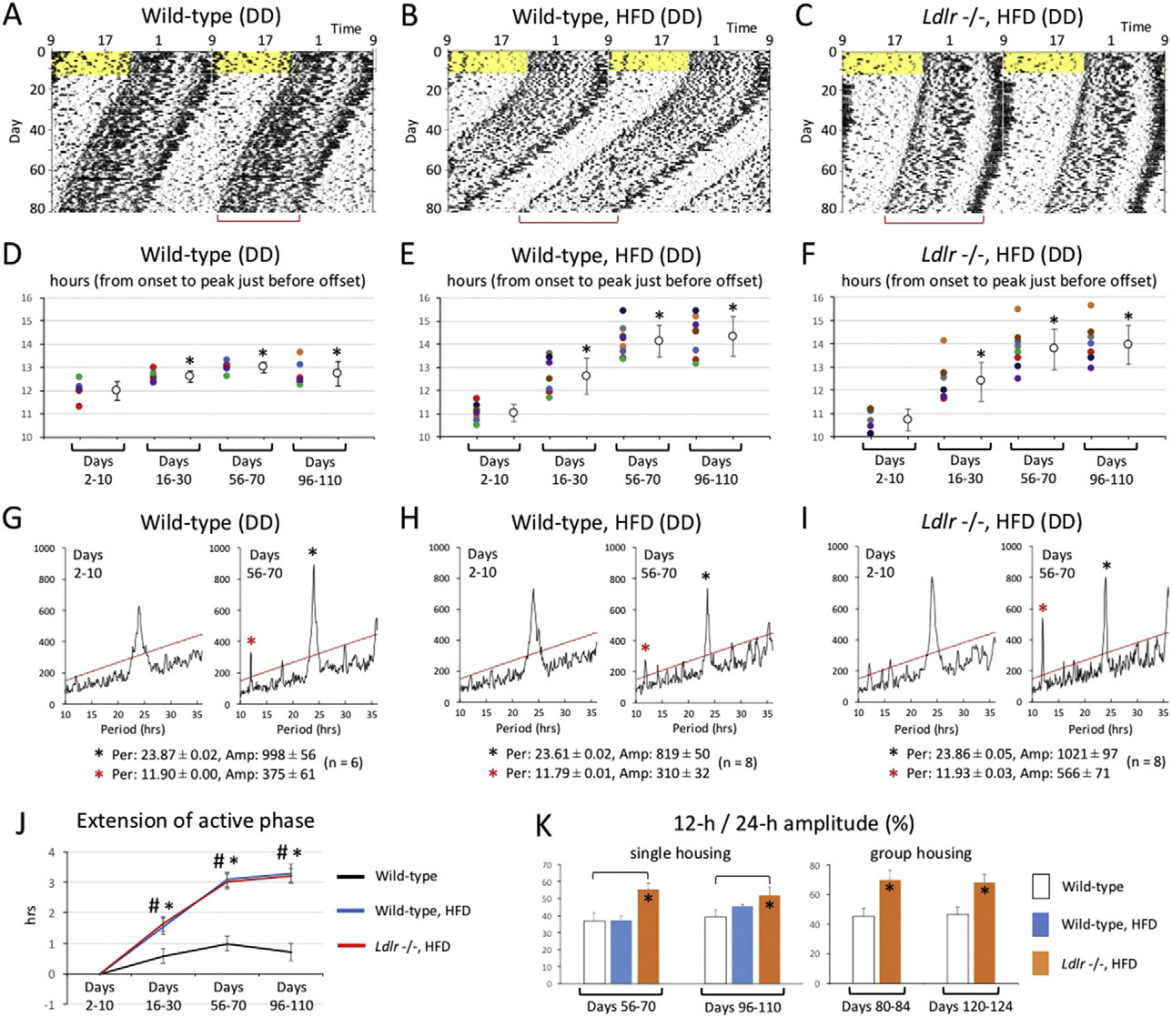
Effect of hypercholesterolemia on behavioral rhythms in constant darkness. (A, B, and C) Continuous monitoring of locomotor activity of isolated mice was performed in constant darkness. Representative raw activity data of wild-type, wild-type (+ HFD) and *Ldlr* −/− (+ HFD) are shown as a double plot. After entrainment to regular light and dark cycles, exposure to the HFD was started on day 11, namely the last day before constant darkness. Yellow shadows indicate light periods. Red square brackets represent active phase duration, whose prolongation was visually observed. (D, E, and F) To calculate average activity over several days by correcting phase difference among days, the time of activity onsets was defined as “time 0”, and then average 6-min bin activity was calculated for several-day periods on days 2–10 (before exposure to the HFD), 16–30 (shortly after exposure to the HFD), 56–70, and 96–110. To compare the duration of the active phase among experimental conditions, the peak time of activity concentrated just before the offset was estimated by non-linear Lorentzian curve fitting. Left dots represent hours from onset to the peak time estimated by Lorentzian curve fitting in individual mice. Right dots indicate the average ± SD (*n* = 6 or 8 mice). A *t*-test (vs. days 2–10) was performed, and significant prolongation of the active phase duration against days 2–10 (before exposure to the HFD) is indicated with asterisks. (G, H and I) The free-running period and amplitude of circadian behavioral rhythms were calculated with a chi-square periodogram on days 2–10 and days 56–70. Graphs indicate a representative of six or eight mice. Red and black asterisks indicate 12-h and 24-h rhythms, respectively. The numbers shown under the periodograms indicate the average ± SE of 24-h/12-h period and amplitude (*n* = 6 or 8 mice). (J) To compare the difference in active phase duration between experimental conditions, the average hour from onset to the peak time estimated by Lorentzian fitting on days 2–10, as calculated in D, E and F, was set to 0. A *t*-test (vs. wild-type) was performed, and a statistically significant difference between wild-type and wild-type (+ HFD) mice, and between wild-type and *Ldlr* −/− (+ HFD) mice, is indicated as asterisks and pound signs, respectively. (K) To examine statistically significant differences in the magnitude of 12-h rhythmicity among experimental conditions, the ratio of 12-h amplitude to 24-h amplitude was calculated in both the single and group housing condition. Data indicate the average ± SE (single: *n* = 6 or 8, group: *n* = 4 or 8). A *t*-test was performed against wild-type mice, and asterisks indicate statistically significant differences.

**Fig. 4:**
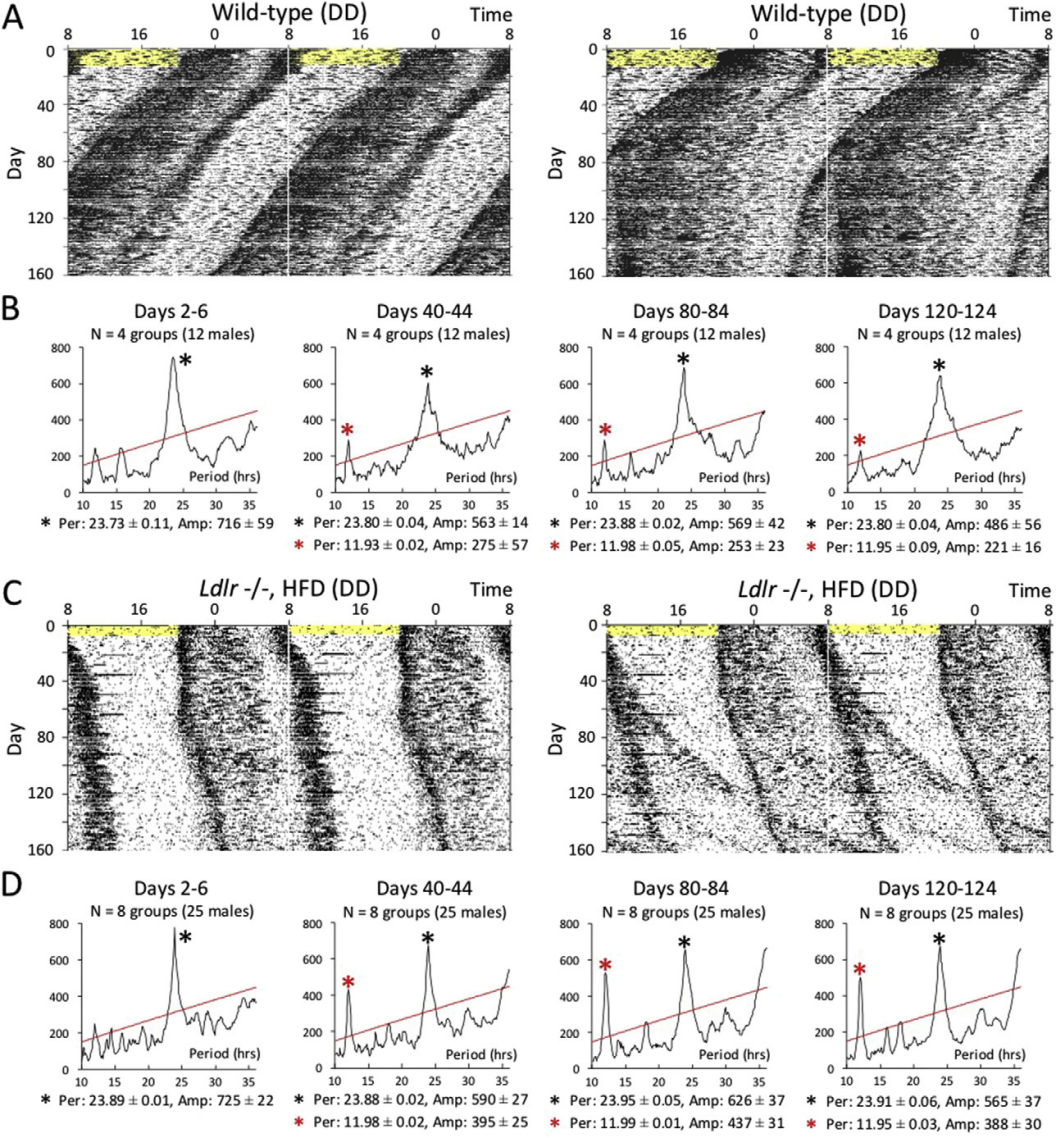
Effect of long-term hypercholesterolemia on behavioral rhythms under constant darkness and group-housing conditions. (A to D) Long-term monitoring of locomotor activity was performed over a period of up to about 160 days in constant darkness. Two representative raw activity data of wild-type mice and *Ldlr* −/− mice (+ HFD) and are shown in (A) and (C), respectively. A few individuals became desynchronized from other animals. The five-day free-running period and amplitude of circadian behavioral rhythms were calculated with a chi-square periodogram at intervals of about 40 days. The data indicate a representative of four wild-type groups (B) and eight *Ldlr* −/− (+ HFD) groups (D). 12-h rhythms (red asterisks) were detectable at days 40–44, 80–84 and 120–124, although only 24-h rhythms (black asterisks) were detectable at days 2–6. The numbers shown under the periodograms indicate the average ± SE of 24-h/12-h period and amplitude (*n* = 4 or 8 groups).

We examined locomotor activity in mice under single-housing and constant darkness conditions (Fig. 3A, B and C). After initial exposure to regular light and dark cycles for 10 days, animals were placed in constant darkness. They were then exposed to the HFD at day 11 that was the first day of constant darkness. We did not examine the effect of hypercholesterolemia on circadian behavior in *Per2* m/m and *Ldlr* −/− *Per2* m/m mice because they showed no behavioral circadian rhythms under constant darkness conditions. Wild-type (+ HFD) and *Ldlr* −/− (+ HFD) mice showed a prolonged active phase after exposure to constant darkness. To compare temporal changes in activity patterns, the time of activity onset was defined as “time 0”, and the average activity level was calculated for several-day periods at four time-points: days 2–10 (before exposure to HFD), days 16–30 (just after exposure to HFD), days 56–70 and days 96–110 (long-term exposure to HFD). To compare active phase duration in wild-type, wild-type (+ HFD), and *Ldlr* −/− (+ HFD) mice, we used a non-linear Lorentzian curve fitting to estimate the peak time of concentrated activity just before offset (Fig. S1 and Fig. 3D, E and F). After exposure to constant darkness, mice in all groups showed a significantly prolonged active phase with time. However, wild-type (+ HFD) and *Ldlr* −/− (+ HFD) mice showed a significantly longer active phase compared to wild-type (− HFD) mice that occurred within a period of 30 days after exposure to constant darkness (Fig. 3J). Since peak intervals of bimodal rhythms appeared to be about 12 h in *Ldlr* −/− (+ HFD) mice, we performed a chi-squared analysis using raw locomotor data (Fig. 3G, H and I). Based on periodograms, we found that although 12-h rhythms were detectable in all three groups, the ratio of the intensity of 12-h amplitude to that of 24-h amplitude was significantly highest in *Ldlr* −/− (+ HFD) mice in both single and group-housing conditions (Fig. 4, Fig. 3K).

Together these results suggest that hypercholesterolemia causes not only an attenuation of onset intensity in light and dark cycles but also distinctly-bimodal behavioral rhythms in constant darkness.

### 3.3 Circadian Dysfunction may be Involved in the Progression of Arteriosclerosis

*Ldlr* −/− mice are well known to develop hypercholesterolemia-mediated arteriosclerosis after exposure to an HFD. Our present behavioral data may indicate that circadian abnormalities are involved in the modification of arteriosclerosis in *Ldlr* −/− mice. To investigate this possibility, we attempted to enhance circadian abnormalities in *Ldlr* −/− mice by introducing a mutation into *Per2*. Both *Ldlr* −/− and *Ldlr* −/− *Per2* m/m mice were fed the HFD to compare the progression of arteriosclerosis between them. LDLR-deficient mice are known to show apparent arterial plaque formation > 16 weeks after HFD treatment although dependent on dietary composition. Additionally, our data based on a pilot experiment in light and dark suggested that the difference in the progression of arteriosclerosis between these mouse strains (*Ldlr* −/− vs. *Ldlr* −/− *Per2* m/m) was not detectable within 20 weeks after HFD treatment (Fig. S2). We therefore decided to perform tissue collection at the 24- and 28-week time points.

**Fig. 5:**
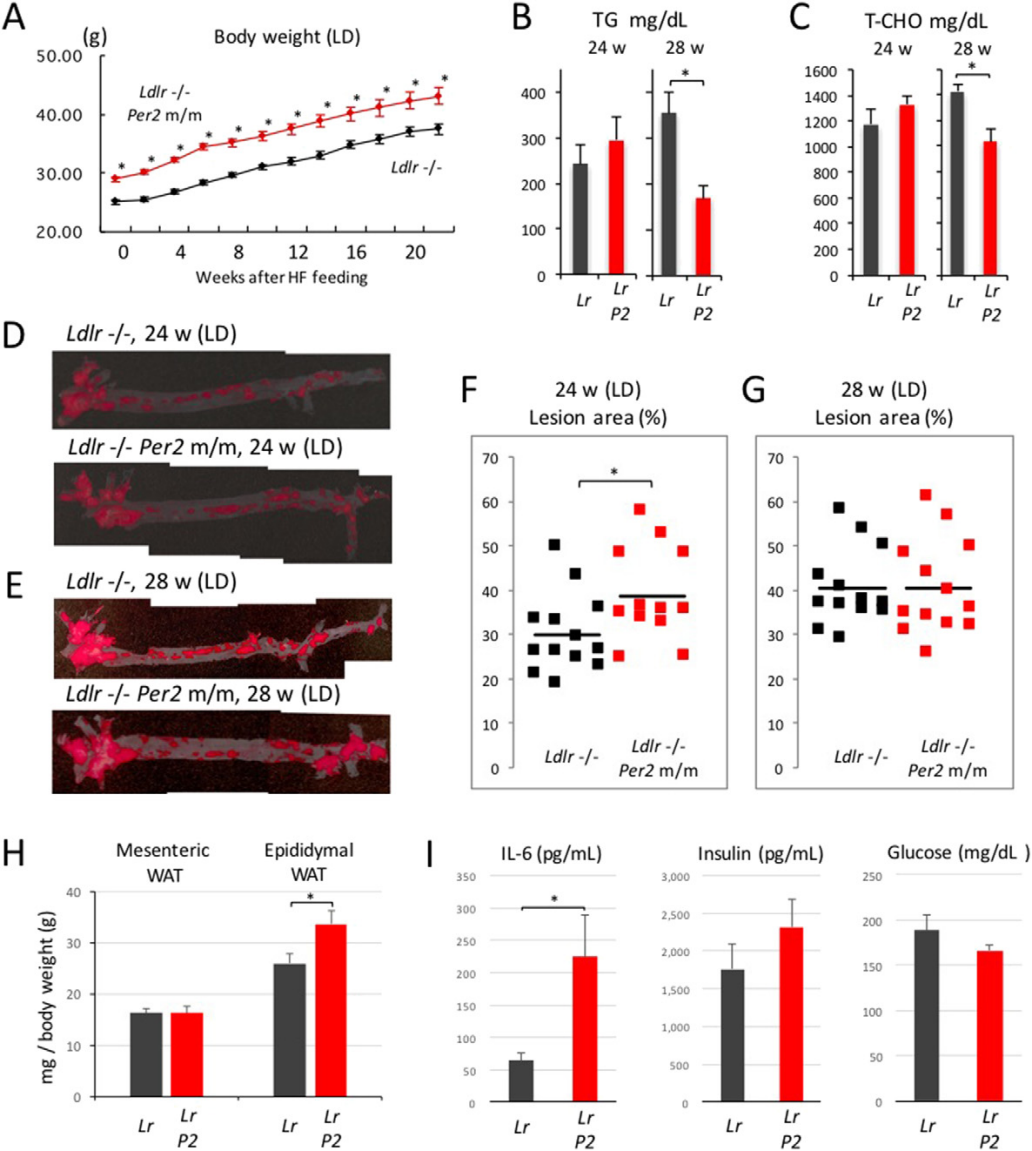
Effect of circadian dysfunction on hypercholesterolemia-induced arteriosclerosis in light and dark. Male *Ldlr* −/− and *Ldlr* −/− *Per2* m/m mice were weaned at 5 weeks of age, exposed to the HFD at 10–11 weeks of age in light and dark, and maintained with the diet for a period of 20, 24 or 28 weeks. (A) Body weight was measured every 2 weeks. The data indicate the average ± SE (*n* = 25 mice). Student's *t*-test (*Ldlr* −/− vs. *Ldlr* −/− *Per2* m/m) was performed and statistical significance was defined as *P < 0.05. (B and C) Mouse blood was collected 24 and 28 weeks after exposure to the HFD, and plasma concentrations of total cholesterol (T-CHO) and triglyceride (TG) were measured. The data indicate the average ± SE (*n* = 12 or 13 mice). Student's *t*-test (*Ldlr* −/− vs. *Ldlr* −/− *Per2* m/m) was performed and statistical significance was defined as *P < 0.05. (D and E) 24 and 28 weeks after exposure to the HFD, aortic samples were fixed in 10% neutral-buffered formalin. Samples were stained in Oil Red O working solution, and then washed with 50% isopropanol. The images indicate representative specimens showing a near-average plaque area 24 and 28 weeks after HFD treatment. (F and G) Image analysis was performed using the ImageJ Software. The area (%) of aortic lesion in each animal was measured as the percentage of lesion area per total area of the aorta. Each dot indicates the lesion area from an individual mouse. The horizontal bold bars indicate the average of 12 (or 13) mice. Student's *t*-test (*Ldlr* −/− vs. *Ldlr* −/− *Per2* m/m) was performed and statistical significance was defined as *P < 0.05. (H) The weight of epididymal and mesenteric white adipose tissues (WAT) was measured and normalized with total body weight at 20 weeks after the start of exposure to the HFD. The data indicate the average ± SE (*n* = 8 mice). A *t*-test was performed and statistical significance was defined as *P < 0.05. (I) The concentration of plasma insulin, glucose, and the representative inflammatory cytokine IL-6 was measured 20 weeks after exposure to the HFD. The data indicate the average ± SE (*n* = 8 mice). A *t*-test was performed and statistical significance was defined as *P < 0.05.

First, we examined the effect of the *Per2* mutation on physiological profiles in light and dark. The body weight of *Ldlr* −/− *Per2* m/m mice was significantly higher than that of *Ldlr* −/− mice at all the time points we measured (Fig. 5A). Since high plasma concentrations of total cholesterol (T-CHO) and triglyceride (TG) are major factors for the development of arteriosclerosis in *Ldlr* −/− mice, we speculated that the introduction of the *Per2* mutation enhanced these levels. Unexpectedly, however, plasma concentrations were decreased rather than increased in these mice (Fig. 5B and C). Next, to visualize and calculate the area of aortic plaque, we performed oil red staining of aortic specimens 24 and 28 weeks after exposure to the HFD. The images indicate representative specimens showing a near-average plaque area 24 and 28 weeks after HFD treatment (Fig. 5D and E). The data demonstrate a significantly larger area of lesion in *Ldlr* −/− *Per2* m/m mice at 24 weeks after HFD treatment (Fig. 5F). After that, *Ldlr* −/− *Per2* m/m mice did not show any further development of aortic plaque, while the lesion area in *Ldlr* −/− mice was enlarged to the same level as *Ldlr* −/− *Per2* m/m mice (Fig. 5G). Taken together, these findings indicate that hypercholesterolemia induced the progression of arteriosclerosis in association with circadian abnormalities.

Given the suggestion that *Per2* mutation-induced progression of arteriosclerosis was not attributable to the increase in plasma lipid concentrations, we examined the possible contribution of other risk factors of arteriosclerosis progression using mice 20 weeks after the start of exposure to the HFD. First, we investigated whether the *Per2* mutation-induced increase in body weight shown in Fig. 5A was caused by the change in adipose tissue weight (Fig. 5H). The weight of epididymal and mesenteric white adipose tissues (WAT) was measured and normalized with total body weight. This showed that introduction of the *Per2* mutation had caused a significant increase in the weight of the epididymal WAT. Arteriosclerosis is a chronic inflammation-related disease, for which an adiposity-induced increase in inflammatory cytokines is well known to be a major risk factor. Next, therefore, we examined the concentration of plasma IL-6, a representative inflammatory cytokine (Fig. 5I), and found an average increase of more than three-fold in *Ldlr* −/− *Per2* m/m mice compared to *Ldlr* −/− mice. Although this result supports our hypothesis, the magnitude of the increase in adipose tissue may not be able to explain this drastic increase in plasma IL-6. In fact, there was no significant difference in adiposity-induced pathologies between these mice when plasma glucose levels and insulin concentrations were measured (Fig. 5I). The drastic increase in IL-6 might therefore involve not only adiposity but also other mechanisms.

**Fig. 6:**
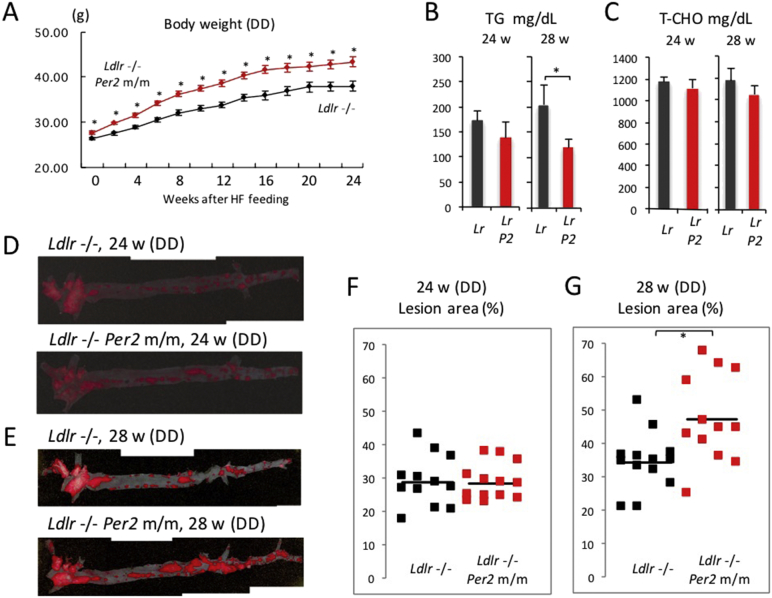
Effect of circadian dysfunction on hypercholesterolemia-induced arteriosclerosis in constant darkness. Male mice were exposed to the HFD at 10–11 weeks of age in constant darkness, and maintained with the diet for a period of 24 or 28 weeks. (A) Body weight was measured every 2 weeks. The data indicate the average ± SE (*n* = 25 mice). Student's *t*-test was performed and statistical significance was defined as *P < 0.05. (B and C) 24 and 28 weeks after exposure to the HFD, plasma concentrations of T-CHO and TG were measured. The data indicate the average ± SE (*n* = 12 or 13 mice). Student's *t*-test was performed and statistical significance was defined as *P < 0.05. (D and E) 24 and 28 weeks after exposure to the HFD, aortic samples were stained in Oil Red O working solution. The images indicate representative specimens showing a near-average plaque area 24 and 28 weeks after HFD treatment. (F and G) The area (%) of aortic lesion in each animal was measured as the percentage of lesion area per total area of the aorta. Each dot indicates the lesion area from an individual mouse. The horizontal bold bars indicate the average of 12 (or 13) mice. Student's *t*-test was performed and statistical significance was defined as *P < 0.05.

Fig. 1 demonstrates that the profile of behavioral rhythms was similar in both *Ldlr* −/− and *Ldlr* −/− *Per2* m/m mice under light and dark conditions. On the other hand, these mice showed behavioral differences in constant darkness. We therefore hypothesized that constant darkness caused a greater difference in plaque formation in these mice. Similarly to the case in light and dark, *Ldlr* −/− *Per2* m/m mice had a significantly higher body weight than *Ldlr* −/− at all the time points we measured (Fig. 6A), and plasma concentrations of total cholesterol and triglyceride in *Ldlr* −/− *Per2* mice were similar or smaller compared to *Ldlr* −/− mice (Fig. 6B and C). Next, we performed oil red staining of aortic specimens 24 and 28 weeks after exposure to the HFD (Fig. 6D and E). The data demonstrate a significantly larger area of lesion in *Ldlr* −/− *Per2* m/m mice at 28 weeks after HFD treatment (Fig. 6F and G). As expected, a slightly larger difference in lesion area between *Ldlr* −/− and *Ldlr* −/− *Per2* m/m mice was detected in constant darkness than in light and dark, although plaque formation speed was lower in constant darkness than in light and dark

## 4 Discussion

Hypercholesterolemia is a common pathology in advanced countries, occurring in 10% to 30% of the population. It is a well-known and major risk factor for a wide range of diseases. The autosomal dominant disorder, familial hypercholesterolemia, is associated with a low density lipoprotein receptor (LDLR) deficiency, and characterized by the presence in children of profound hypercholesterolemia, cutaneous planar xanthomas, and rapidly progressive coronary vascular disease that usually results in death before age 30 years (Francke et al., 1984, Goldstein and Brown, 1973). We conducted the present study to investigate whether hypercholesterolemia is involved in the triggering of circadian abnormalities, which are increasingly common in advanced countries and which cause circadian-related pathologies (Roenneberg et al., 2012).

To examine this hypothesis, we used mice lacking functional LDLR, an animal model for hypercholesterolemia. We found that free-running rhythms in locomotor activity began to show a distinctly bimodal pattern like a 12-h ultradian rhythm after exposure to constant darkness. Even when the circadian clock was entrained by light and dark cycles, behavioral onset intensity at the start of the dark phase was significantly attenuated by hypercholesterolemia. A previous study reported that an HFD resulted in the damping of peripheral clock gene expression rhythms, and that these mice showed a significantly decreased amplitude and a longer free-running period in locomotor activity rhythms (Kohsaka et al., 2007), but did not show abnormalities such as onset intensity attenuation or distinctly bimodal rhythmicity, which were seen in *Ldlr* −/− mice. As an interpretation of the different phenotypes in HFD-fed mice in comparison with *Ldlr* −/− mice, HFD treatment of wild-type mice is known to be insufficient to cause a severe increase in plasma cholesterol concentrations. However, as a limitation of the present study, although wild-type mice were littermates for *Ldlr* −/− mice and *Ldlr* −/− mice had been repeatedly backcrossed with C57BL/6 mice (recipient genome > 99.99%), we cannot exclude the possibility that the 0.01% genetic background difference between wild-type and *Ldlr* −/− mice could influence the free-running behavior. Contradicting the previous finding that a HFD lengthened circadian period in wild-type mice, our data indicate that period length is shorter under HFD conditions (compare Fig. 3A with Fig. 3B). We consider that the reason for the different effect of an HFD on period length is due to different experimental conditions. While an HFD used in the previous report contained 45% kcal from fat, 20% kcal from protein and 35% kcal from carbohydrate, the HFD in the present study contains 36.9% kcal fat, 20.4% kcal protein and 42.7% kcal carbohydrate; in other words, fat is 8.1% less than that in the previous study. Additionally, the start time of exposure to the HFD is largely different between them: while exposure started 14 days after the onset of DD in the previous study, it started at the same time as the onset of DD in the present study.

There are several potential molecular pathways via which hypercholesterolemia-induced chronic metabolic abnormalities might affect circadian profiles, as follows. First, the DNA-binding activity of the BMAL1 and CLOCK heterodimer is regulated by the redox state of nicotinamide adenine dinucleotide (NAD) cofactors (Rutter et al., 2001). Indeed, an HFD causes the impairment of CLOCK-BMAL1 chromatin recruitment (Eckel-Mahan et al., 2013). Second, SIRT1-mediated deacetylation of BMAL1, PER2 and the coactivator PGC-1a affects circadian clock gene expression (Asher et al., 2008, Chang and Guarente, 2013, Nakahata et al., 2008). Third, the nutrient-responsive adenosine monophosphate-activated protein kinase (AMPK) phosphorylates and destabilizes CRY1 (Lamia et al., 2009). These molecular pathways might be activated or suppressed in response to chronic intracellular metabolic changes induced by hypercholesterolemia, leading to behavioral abnormalities. To date, although no data supportive of their involvement in abnormal behavior of *Ldlr* −/− mice has yet been obtained, our experimental data using *Per2* m/m (*Ldlr* +/+) mice in light and dark in Fig. 1 might indicate that the effect of chronic hypercholesterolemia on behavioral rhythms involve malfunction of the core clock machinery.

If these results are translatable to humans, chronic hypercholesterolemia may cause a reduction in physical and mental performance during the morning, leading to a decline in the quality of social life (Fig. S3). In addition, abnormal circadian rhythms might exacerbate the misalignment of internal and social rhythms, likely leading to a wide range of circadian-related diseases. Efforts to slow or stop the recent rapid increase in circadian misalignment in advanced countries might therefore necessarily require the control of plasma cholesterol concentrations.

Further, we hypothesized that circadian abnormalities might affect the progression of hypercholesterolemia-related diseases. For example, the arteriosclerosis shown in *Ldlr* −/− mice might be at least partially mediated by circadian dysfunction. To examine this possibility, we generated *Ldlr* −/− mice with more severely impaired circadian rhythms by introducing a mutation into *Per2*, and as expected, we found that these mice showed a significant enlargement of artery plaque area. With regard to why the difference between mouse strains appeared more slowly in DD than in LD as shown in Fig. 5, Fig. 6, our interpretation is as follows. Results of behavioral analysis using mice in DD indicated that *Ldlr* −/− mice treated with the HFD showed an extended active period and behavioral splitting, while *Ldlr* −/− *Per2*m/m mice treated with the HFD showed a short circadian period during the early experimental period and then arrhythmic behavior. Because these abnormal circadian characteristics made it difficult for these mice to adapt regular LD cycles, arterial plaque formation may be differently affected by chronic forced adaptation to the environment compared to the free-running condition (DD). As another example that the difference between internal and external rhythmicity causes homeostatic dysfunction, a very recent study using *Cry* KO mice reported that the difference between internal and external period lengths causes irregular estrous cycles in mice (Takasu et al., 2015). However, to fully resolve this issue, an experiment such as forced desynchrony or an LD schedule which exceeds the limits of circadian entrainment is necessary.

Although high plasma concentrations of total cholesterol and triglyceride are major factors in the development of plaque in *Ldlr* −/− mice, they were unexpectedly decreased rather than increased in *Ldlr* −/− *Per2* m/m mice. Hypertension is also a known risk factor for the progression of arteriosclerosis. However, it was previously reported that the *Per2* mutation does not affect blood pressure levels (Vukolic et al., 2010). Interestingly, we found that the *Per2* mutation promoted adiposity, another risk factor for arteriosclerosis. A previous report suggested that PER2 functions as a repressor of adipogenesis via PPARγ in vitro, albeit that adiposity was not observed in *Per2*-deficient mice in vivo (Grimaldi et al., 2010). Our present results indicate that adiposity was observed in *Ldlr* −/− *Per2* m/m mice when compared to *Ldlr* −/− mice, which in turn indicated that hyperlipidemia invoked the repressive function of PER2 against adipogenesis in vivo. Arteriosclerosis is a chronic inflammation-related disease, and the increase in inflammatory cytokines induced by adiposity is well known to be a major risk factor for arteriosclerosis. We therefore examined the concentration of plasma IL-6, a representative inflammatory cytokine, and found that it increased more than three-fold on average in *Ldlr* −/− *Per2* m/m mice when compared to *Ldlr* −/− mice. However, the magnitude of the increase in adipose tissue in our present experiments may not be able to sufficiently explain this drastic increase in plasma IL-6. In this context, a few reports have suggested that the circadian synthesis of IL-6 and phase-responsiveness of IL-6 to endotoxin in macrophages are controlled by the cell-autonomous circadian clock, and that IL-6 expression is potently inhibited by the clock component REV-ERBα (Keller et al., 2009) (Gibbs et al., 2012), whose expression is highly reduced in *Per2*-mutant mice (Preitner et al., 2002). Given these previous findings, the *Per2* mutation-induced increase in plasma IL-6 may be caused not only by adiposity-triggered inflammation but also by decreased REV-ERBα expression in macrophages, leading to modification of arteriosclerosis (Fig. S3).

On the other hand, a comprehensive gene expression analysis revealed that > 300 transcripts exhibited a circadian pattern of oscillation in mouse aorta, indicating that the circadian clock potently regulates a wide range of vascular function (Rudic et al., 2005). Consistent with this, it has been reported that the *Per2* mutation results in vascular endothelial dysfunction as follows. First, a previous report indicated that *Per2*-deficient mice showed aortic endothelial dysfunction involving decreased production of NO and vasodilatory prostaglandins and increased release of vasoconstrictors (Viswambharan et al., 2007). Second, it was reported that *Per2* mutation caused vascular senescence and impaired ischemia-induced revascularization (Wang et al., 2008). Thus, circadian abnormalities induce vascular dysfunction, which may contribute to the progression of hypercholesterolemia-induced arteriosclerosis.

Taken together, our findings indicate that hypercholesterolemia induces circadian abnormalities, leading to a decline in the quality of social life and an increase in the risk of circadian-related diseases. Further, the diversification and progression of hypercholesterolemia-related diseases might be partially in association with circadian dysfunction (Fig. S3).

## Funding Sources

This work was supported by fellowships from the Japan Heart Foundation (Novartis Grant for Research Award on Molecular and Cellular Cardiology and Pfizer Grant for Research on Hypertension, Hyperlipidemia and Vascular Metabolism), the SENSHIN Medical Research Foundation and the Japan Society for the Promotion of Science.

## Author Contributions

M.A. conceived and supervised the project, performed experiments, analyzed data, and wrote the manuscript. R.M. analyzed data. T.M., Y.K. and H.K. performed experiments. K.N. provided general supports and gave conceptual advice.

## Conflicts of Interest

The authors declare no competing financial interests.

## Appendix A Supplementary Data

Supplementary figures Image 1
