# Supplementary material for: Hypercholesterolemia Causes Circadian Dysfunction: A Potential Risk Factor for Cardiovascular Disease
Source: eBioMedicine. 2017 Apr 27;20:127–36. doi: 10.1016/j.ebiom.2017.04.034 (PMC5478205; doi:10.1016/j.ebiom.2017.04.034)
Supplement: Supplementary file 1 — Supplementary figures [file mmc1.pdf]

Supplemental Figures

Figure S1

Examples of the results obtained using a non-linear Lorentzian curve fitting

To calculate average activity over several days by correcting phase difference among days, the time of activity onsets was defined as "time 0", and then average 6-min bin activity was calculated for several-day periods (for an example, days 56-70). And then, to compare the duration of the active phase among experimental conditions, the peak time of activity concentrated just before the offset was estimated by non-linear Lorentzian curve fitting.

Figure S2

Effect of circadian dysfunction on hypercholesterolemia-induced arteriosclerosis in light and dark cycles at 20 weeks after exposure to the HFD

Male *Ldlr* -/- and *Ldlr* -/- *Per2* m/m mice were exposed to the HFD at 10-11 wk of age in light and dark, and maintained with the diet for a period of 20 weeks. (A) Body weight was measured at indicated time points. The data indicate the average ± SE (n = 8 mice). A t-test was performed and statistical significance was defined as *P < 0.05. (B and C) Mouse blood was collected 20 weeks after exposure to the HFD, and plasma concentrations of total cholesterol (T-CHO) and triglyceride (TG) were measured. The data indicate the average ± SE (n = 8). The average concentrations in *Ldlr* -/- mice were set to 100%. A t-test was performed and statistical significance was defined as *P < 0.05. (D) 20 weeks after exposure to the HFD, aortic samples were fixed in 10% neutral-buffered formalin, and then stained in Oil Red O working solution. Image analysis was performed using the ImageJ Software. The area (%) of aortic lesion in each animal was measured as the percentage of lesion area per total area of the aorta. Each dot indicates the lesion area from an individual mouse. The horizontal bold bars indicate the average of eight mice. A t-test was performed, but statistical significance (P < 0.05) was not achieved between *Ldlr* -/- and *Ldlr* -/- *Per2* m/m mice.

Figure S3

Schematic summary of the relationship between hypercholesterolemia and circadian abnormalities

Figure S1

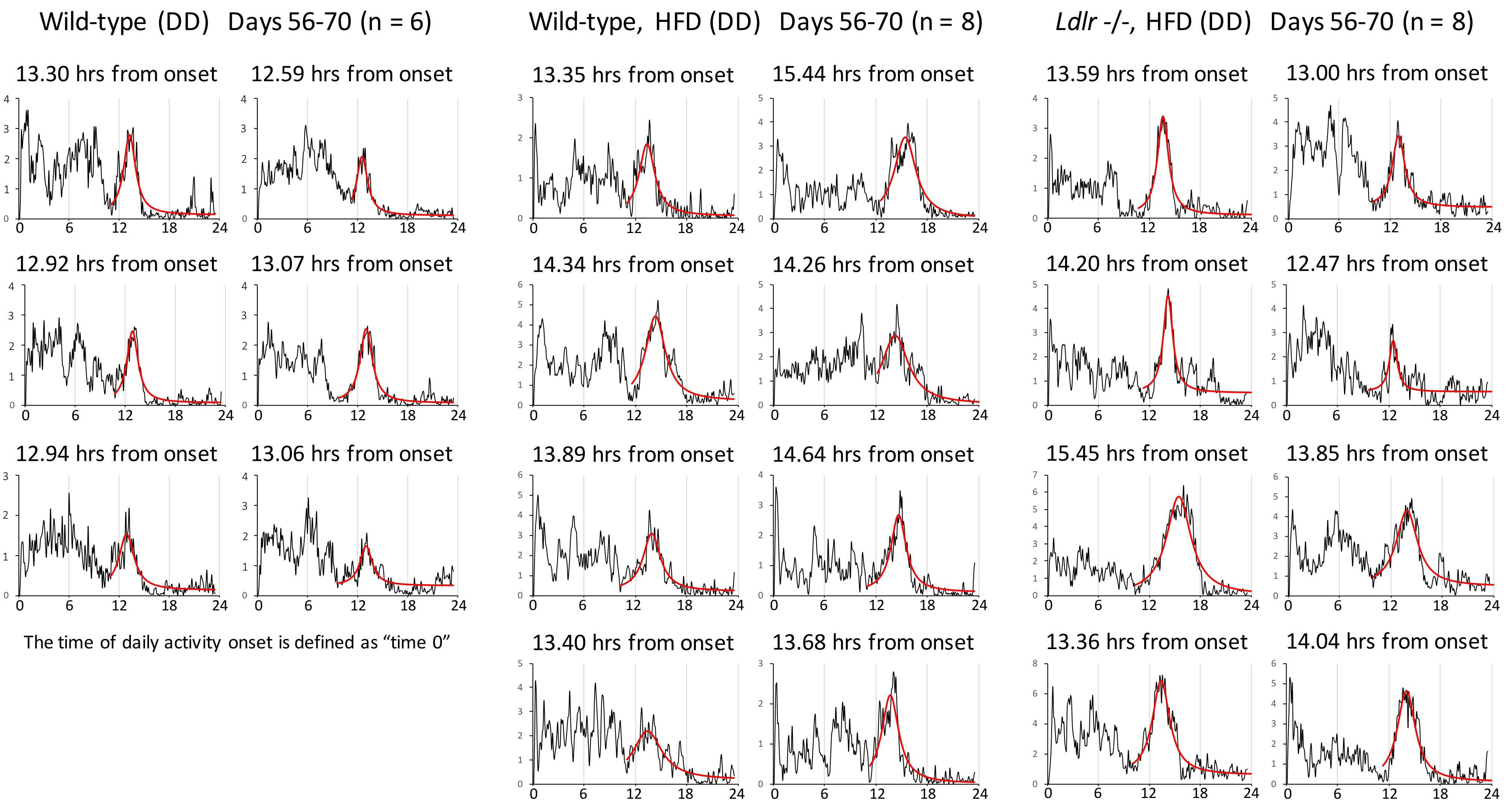

Wild-type (DD) Days 56-70 (n = 6)
Wild-type, HFD (DD) Days 56-70 (n = 8)
Ldlr -/-, HFD (DD) Days 56-70 (n = 8)
13.30 hrs from onset
12.59 hrs from onset
13.35 hrs from onset
15.44 hrs from onset
13.59 hrs from onset
13.00 hrs from onset
12.92 hrs from onset
13.07 hrs from onset
14.34 hrs from onset
14.26 hrs from onset
14.20 hrs from onset
12.47 hrs from onset
12.94 hrs from onset
13.06 hrs from onset
13.89 hrs from onset
14.64 hrs from onset
15.45 hrs from onset
13.85 hrs from onset
13.40 hrs from onset
13.68 hrs from onset
13.36 hrs from onset
14.04 hrs from onset
The time of daily activity onset is defined as “time 0”

Figure S2

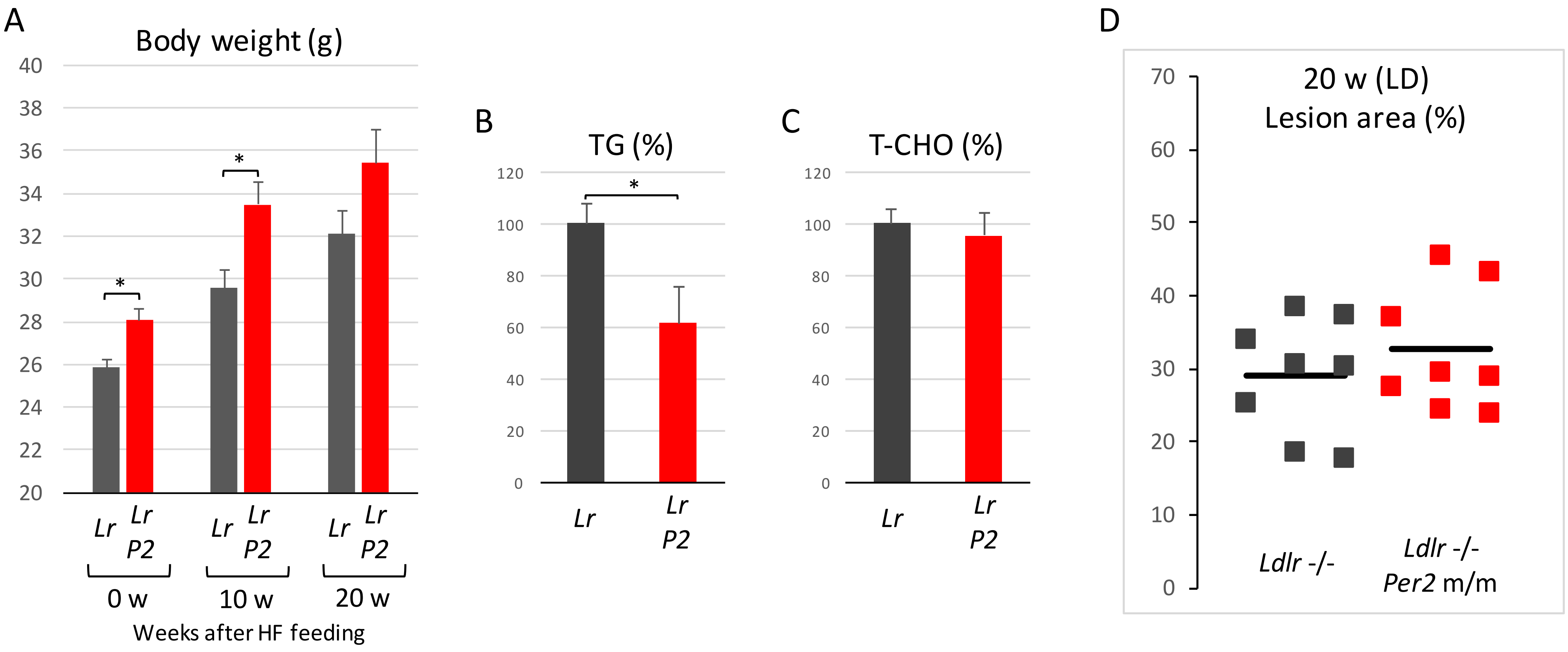
A
Body weight (g)
40
38
36
34
32
30
28
26
24
22
20
*
*
Lr
Lr P2
Lr
Lr P2
Lr
Lr P2
0 w
10 w
20 w
Weeks after HF feeding
B
TG (%)
120
100
80
60
40
20
0
*
Lr
Lr P2
C
T-CHO (%)
120
100
80
60
40
20
0
Lr
Lr P2
D
20 w (LD)
Lesion area (%)
70
60
50
40
30
20
10
0
Ldlr -/-
Ldlr -/- Per2 m/m

Figure S3

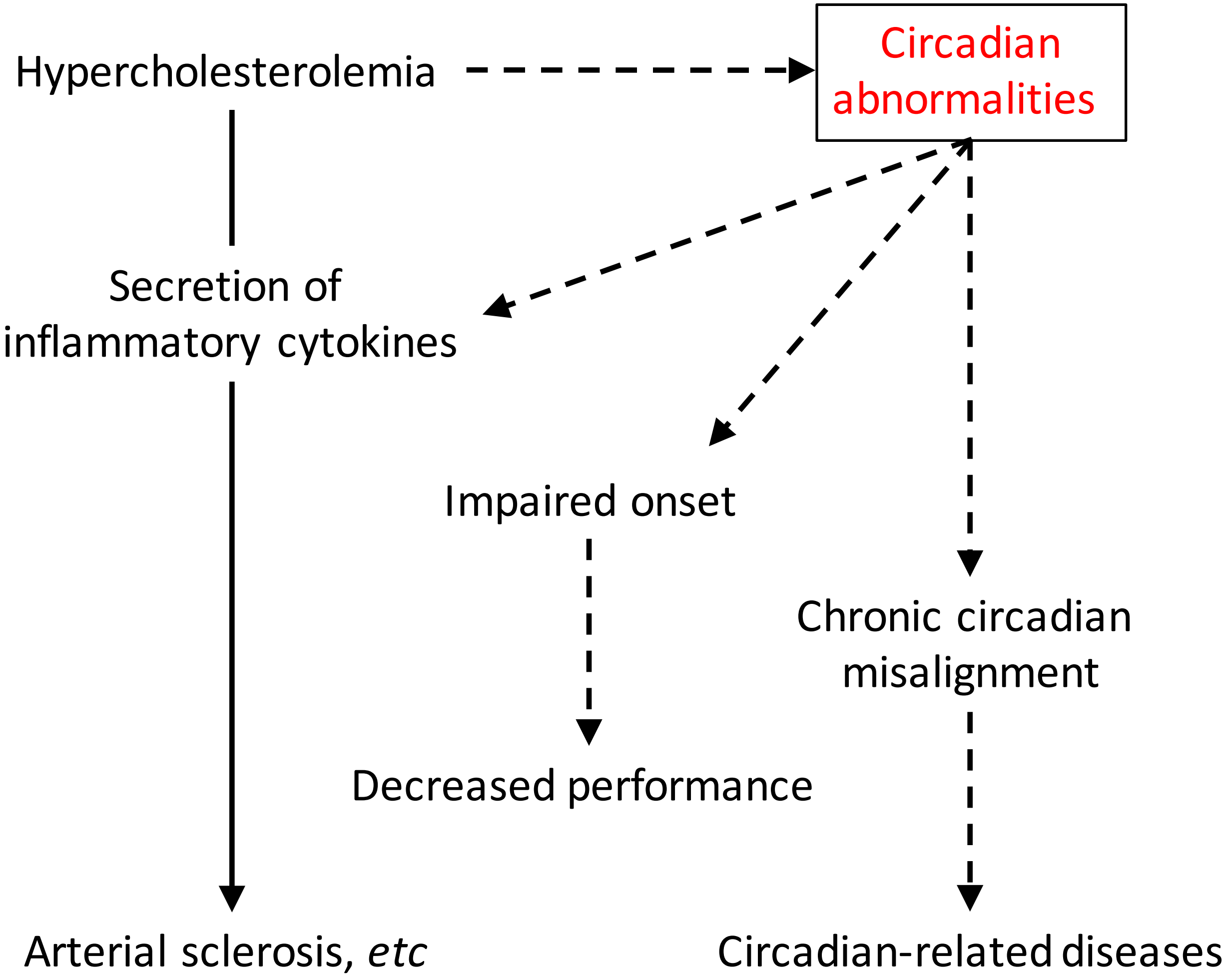
Hypercholesterolemia
Circadian abnormalities
Secretion of inflammatory cytokines
Impaired onset
Chronic circadian misalignment
Decreased performance
Arterial sclerosis, *etc*
Circadian-related diseases
